# Deafness gene screening based on a multilevel cascaded BPNN model

**DOI:** 10.1186/s12859-023-05182-7

**Published:** 2023-02-20

**Authors:** Xiao Liu, Li Teng, Wenqi Zuo, Shixun Zhong, Yuqiao Xu, Jing Sun

**Affiliations:** 1 School of Microelectronics and Communication Engineering, Chongqing University, 174 Shapingba District, Chongqing, 400044 China; 2 Department of Otolaryngology, The First Affiliated Hospital of Chongqing Medical University, NO. 1 Youyi Road, Yuzhong District, Chongqing, 400016 China

**Keywords:** Sudden sensorineural hearing loss, Backpropagation neural network, Cascaded BPNN model, Highly suspected deafness-related genes

## Abstract

Sudden sensorineural hearing loss is a common and frequently occurring condition in otolaryngology. Existing studies have shown that sudden sensorineural hearing loss is closely associated with mutations in genes for inherited deafness. To identify these genes associated with deafness, researchers have mostly used biological experiments, which are accurate but time-consuming and laborious. In this paper, we proposed a computational method based on machine learning to predict deafness-associated genes. The model is based on several basic backpropagation neural networks (BPNNs), which were cascaded as multiple-level BPNN models. The cascaded BPNN model showed a stronger ability for screening deafness-associated genes than the conventional BPNN. A total of 211 of 214 deafness-associated genes from the deafness variant database (DVD v9.0) were used as positive data, and 2110 genes extracted from chromosomes were used as negative data to train our model. The test achieved a mean AUC higher than 0.98. Furthermore, to illustrate the predictive performance of the model for suspected deafness-associated genes, we analyzed the remaining 17,711 genes in the human genome and screened the 20 genes with the highest scores as highly suspected deafness-associated genes. Among these 20 predicted genes, three genes were mentioned as deafness-associated genes in the literature. The analysis showed that our approach has the potential to screen out highly suspected deafness-associated genes from a large number of genes, and our predictions could be valuable for future research and discovery of deafness-associated genes.

**Supplementary Information:**

The online version contains supplementary material available at 10.1186/s12859-023-05182-7.

## Introduction

Sudden deafness, also known as sudden sensorineural hearing loss (SSNHL), is a kind of hearing impairment syndrome characterized by sudden occurrence, unknown cause and rapid development within seconds to days. SSNHL is generally defined as 30 decibels or more sensorineural hearing loss with at least three consecutive frequencies within 72 h [1–5]. Other definitions include hearing loss within 12 or 24 h to emphasize the concept of suddenness [6, 7]. It has been reported that 5 to 20 out of 100,000 people worldwide suffer from SSNHL every year. The disease can occur at any age, but the highest incidence is among individuals aged 40–60 years [8].

Some researchers believe that sudden deafness is associated with mutations in hereditary deafness genes. Chen et al. found experimentally that the homozygous GJB2 c.109G > A mutation may be a cause of sudden deafness involving both ears [9]. Gross et al. found that the MTHFR C677T mutation is associated with an increased risk of SD, which appears to be independent of blood folic acid and homocysteine levels [10]. Uchida et al. concluded that the T allele of MTHFR C677T could be associated with susceptibility to SSNHL and even implied that this mutation could be a risk factor that is independent of blood folic acid and homocysteine [11]. Hamidi et al. found that the MTHFR C677T and ApoE gene variants may be associated with sudden sensorineural hearing loss in an Iranian population [12]. Furuta et al. found that interleukin-1 gene (*IL1A*) polymorphisms were closely related to SSNHL and Meniere's disease using controlled experiments and statistical analysis [13]. Yang et al. used real-time quantitative reverse transcription-polymerase chain reaction (qRT‒PCR) to detect that *TLR2* expression was closely related to the severity of SNNHL [14]. Cao et al. conducted a systematic review of the causes of sudden deafness in recent years, and a large number of studies support the association of genetic polymorphisms with SSNHL susceptibility [15].

Many scholars have investigated the etiology, diagnosis, treatment and prognosis of sudden deafness [16–21]. Most studies used sudden deafness patients as the experimental group and normal hearing people as the control group. Patient DNA was extracted and sequenced by methods such as Sanger sequencing, second-generation sequencing, and third-generation genome sequencing with PCR amplification technology. Then, SPSS was used to analyze the correlations between gene mutation sites and sudden deafness [12–14, 22–24]. Although this experiment-based method is highly accurate, it is expensive, time-consuming, and laborious, especially at the sequencing step, making it unsuitable for universal use in the diagnosis of sudden deafness patients.

Currently, machine learning-based classification algorithms have been utilized to predict and identify disease genes. The gene sequences are obtained by finding the genes corresponding to the diseases in the database, and the corresponding gene features are extracted by using the disease similarity network, gene-phenotype similarity network and gene expression data, etc. The extracted features are used to train the classifier and to predict and classify the genes [25–27]. Azadi et al. used a graph-based correlation-redundancy gene selection approach for cancer diagnosis [28]. Saberi et al. combined matrix decomposition and minimum redundancy based bi-regular unsupervised feature selection was applied to gene selection [29]. Building on traditional machine learning models, cascading basic models have been used to explore diseases. For example, Guo et al. proposed a BCD forest model, a boosting cascade deep forest model for the classification of cancer subtypes based on gene expression data [30]. Su et al. used a deep forest model to predict anticancer drug response [31]. In the past few years, researchers have begun to use machine learning algorithms to detect and identify hearing loss in sudden deafness. For example, Bing et al. used machine learning models to predict hearing outcomes in sudden sensorineural hearing loss [32], and Deepak et al. proposed a Jaya algorithm based on mutation and limit learning machines for sensorineural hearing loss detection [33]. The focus of these studies has been mainly on studying hearing loss in deaf patients without further studies on the genes associated with sudden neurological hearing loss. Here, we proposed a machine learning approach to identify candidate highly suspicious deafness genes. Three basic BPNN models were cascaded to constitute a cascaded BPNN model, which has a stronger ability for screening deafness-associated genes than the conventional BPNN model. Since there is no database to collect data on genes related to sudden deafness, a large number of studies have shown that sudden deafness is closely related to hereditary deafness genes. In this paper, we compiled research reports on sudden deafness from the Web of Science and Engineering Village and searched genetic deafness gene databases such as the Deafness Variation Database v9.0 (DVD).

In this study, 211 of 214 deafness-related genes in DVD [34] were used as positive data, and 2,110 genes extracted from chromosomes were used as negative data to train our model. A total of 80 features were used to describe the deafness-associated genes, including sequence-based features, protein-based features, Hurst index, and information-theoretic features. To test the effectiveness of the model, 45 determined deaf genes were collected from the literature, and three genes from the Fifteen Deafness-Related Gene Mutations Detection Kit were classified separately. The average AUC of the experimental results was above 0.94, which indicates the potential of our model to assist in screening highly suspected deafness-related genes from a large number of genes.

Moreover, to illustrate the predictive performance of the model on suspected deafness-related genes, we analyzed and scored the remaining 17,711 genes (the approximately 20,035 genes in the human genome minus the genes used in the previous experiment). The top 20 scored genes were labeled highly suspected deafness-related genes. We found that three of the top 20 genes mentioned in the literature were deafness-related genes (the top 100 scored genes are listed in Additional file 1: Table A.1). The results show the potential of our cascaded classification model for screening highly suspected deafness-related genes from a large number of genes. We proposed that this model could be used to screen highly suspected deafness-related genes and provide valuable guidance for the clinical diagnosis and treatment of sudden deafness.

## Materials and methods

All analyses were performed on an Intel I7-7770 (3.6 GHz) computer with 16 GB memory, and the whole process described in the paper was implemented in a 64-bit Python 3.7 platform.

**Fig. 1:**
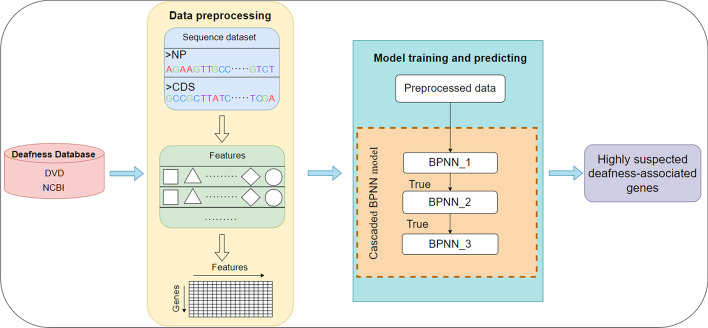
Overview of our approach

Figure 1 illustrates the overview of the proposed method for the prediction of highly suspected deafness-related genes.

### Deafness gene collection and preprocessing

The deafness-related gene data were obtained from DVD and the National Center of Biotechnology Information (NCBI). A total of 211 deafness-related genes were downloaded from the DVD database. All genes from the human genome were downloaded from NCBI. The corresponding gene coding sequences (CDSs) and protein sequences were also obtained from NCBI. The data from NCBI were cleaned to remove duplicates.

A gene may have multiple gene CDSs, and each gene CDS corresponds to a protein sequence. Each sample represents a combination of the features of a gene CDS and the features of the corresponding protein sequence. Therefore, the number of samples is equal to the number of gene CDSs.

### Positive data

The positive data consisted of 571 gene CDSs and 571 protein sequences of the 211 deafness-related genes from the DVD (see Table 1).

**Table 1:** Description of the experimental dataset

Data set	Number of genes	Number of gene CDSs	Number of protein sequences	Number of samples
Positive(P)	211	570	570	570
Negative(N)	210	570	570	570
Test bench 1	45	100	100	100
Test bench 2	3	3	3	3
Test bench 3	17,711	26,436	26,436	26,436

### Negative data

It is difficult to determine the nondeafness-related genes (negative set), as there is no database or article that clearly indicates which gene locus mutations are completely unrelated to deafness. There are 20,035 genes in the human genome. Given the three deafness-related genes in the Detection Kit used as test data (see description of test bench 2) and the 211 genes used as positive data, there were 17,711 genes left. According to the proportion of the 211 deafness-related genes in corresponding human chromosomes, we randomly extracted 2110 genes from the 19,883 genes. These 2110 genes were not among the deafness-related genes mentioned in the literature extracted from Web of Science, EI and other databases. In this study, we used these 2110 genes as nondeafness-related genes. Their gene CDSs and protein sequences were downloaded, and duplicate sequences were removed. Then, we obtained 4945 gene CDSs and 4945 protein sequences as negative data. In each experiment, 571 samples were randomly selected from the 4945 samples to make a negative set. The ratio of the data in the positive set to the data in the negative set was 1:1 (Table 1).

There are two reasons why we constructed the negative set using this strategy. First, the number of potential undiscovered deafness-related genes is far less than the number of nondeafness-related genes, and the potential deafness-related genes have only a small chance of being selected as nondeafness-related genes [35]. Second, each cascaded basic BPNN model was subjected to 50 experimental cycles to improve the stability and accuracy of the model and reduce the possibility of selecting potential deafness-associated genes as nondeafness-associated genes in the experiment.

### Test bench dataset

To demonstrate the accuracy and validity of the model, we designed and analyzed three test bench datasets for evaluation.

Test bench dataset 1 contained 45 deafness-related genes, including 100 gene CDSs and protein sequences. Test bench dataset 1 was used as a test dataset for evaluation classification with the trained model.

Test bench dataset 2 contained three genes from the Fifteen Deafness-Related Gene Mutations Detection Kit (Microarray), which is widely used in China (registration number 20173401343 in the China National Medical Products Administration). The kit was used for the detection of 15 mutation sites. There is one more *mitochondrial 12S rRNA* listed in the detection kit for clinical diagnosis. However, its gene CDS and protein sequence were not obtained; therefore, we removed it. Then, three genes (i.e., *SLC26A4*, *GJB2*, and *GJB3*) were used to construct test bench dataset 2 as a dataset closely related to clinical diagnosis for evaluation classification with the trained model. (Such as Table 1).

Test bench dataset 3 contains the remaining 17,711 genes—those remaining in the human genome after excluding those already involved in the experiment; we considered them unidentified genes and evaluated the top 20 highly suspected deafness-related genes. The top 100 scored genes are listed in Additional file 1: Table A.1.

To improve the universality of the model and avoid overfitting, in the experiment, the dataset was divided into a training set, a validation set and a test set in a 6:2:2 ratio. The training set (ratio of positive and negative data 1:1) was used to train the model, the validation set was used to adjust the parameters of the model, and the test set was extracted separately before the model training to test the performance of the model.

### Feature extraction

In studies of the computational prediction of disease genes, researchers have proposed a variety of related features, including high-throughput experimental features, protein‒protein interaction data, or gene-expression data [36–42]. However, many of these features are based on experimental data, which are not easy to obtain. The experimental data-based features of new genomes are also generally absent, resulting in a limited scope of computational prediction applications. To address this issue, we focused on sequence-based features. The feature set we selected included 80 features: 2 inherent features, 13 codon bias features, 22 amino acid use frequency features, 12 amino acid physicochemical property features, 3 transmembrane helix-like features, the Hurst index and 26-dimensional information entropy features. These features were calculated by bioinformatics tools and Python 3.7, as shown in Table 2.

**Table 2:** Description of the features used in the experiment

Type	Feature	Feature description	Tools
Inherent feature	CDS size	Gene coding sequence length	Python 3.7
	Protein size	Amino acid length	
CodonW	T3s, C3s,	Relative synonymous codon usage of	CodonW [43]
	A3s, G3s	T, C, A, and G at the 3^rd^ position	
	CAI	Codon adaptation index	
	CBI	Codon bias index	
	Fop	Frequency of optimal codons	
	Nc	Effective number of codons	
	GC3s	GC of silent 3rd codon posit	
	GC	GC content of gene	
	L_sym	Number of synonymous codons	
	Gravy	Hydrophobicity of protein	
	Aromo	Aromaticity of protein	
Amino acid usage frequency	Amino acid	A,R,D,C,Q,E,G,H,I,N,L,K,M,F,P,S,T,W,Y,V	Python 3.7
	Rare_aa_ratio	Frequency of rare amino acids	
	Close_aa_ratio	Number of codons 3rd stop codon mutation	
Physicochemical properties of amino acids	M_weight	Molecular weight	Pepstats [44]
	I_Point	Isoelectric point	
	Tiny	(A + C + G + S + T)	
	Small	(A + B + C + D + G + N + P + S + T + V)	
	Aliphatic	(A + I + L + V)	
	Aromatic	(F + H + W + Y)	
	Nonpolar	(A + C + F + G + I + L + M + P + V + W + Y)	
	Polar	(D + E + H + K + N + Q + R + S + T + Z)	
	Charged	(B + D + E + H + K + R + Z)	
	Basic	(H + K + R)	
	Acidic	(B + D + E + Z)	
	A_R Weight	Average Residue Weight	
Transmembrane helix	ExpAA	Exp number of AAs in TMHs	TMHMM3 [45]
	First60	Exp number, first 60 AAs	
	PredHel	Total prob of N-in	
Hurst	Hurst	Hurst index	R package [46]
Information Entropy	Shannon Entropy	quantifies the average information content of the gene sequence from the distribution of symbols	Python 3.7
	Mutual Information	measures the information shared by two random variables	Python 3.7
	Kullback–Leibler divergence	measure the similarity of two probability distributions	Python 3.7
	Cross Entropy	measure the difference information between two probability distributions	Python 3.7

### Shannon entropy

Shannon entropy is widely used in gene expression analysis in bioinformatics. There are differences in the conservation and correlation between different regions and sites in the DNA sequence, resulting in different information entropy values. In this work, we used Shannon entropy to analyze sequence regions and sites. We first digitized the DNA sequence according to the method that the four nucleotides, A, G, C, and T, were assigned the digital numbers 0, 1, 2, and 3, respectively, and then we used the calculation formula of Shannon entropy [47]. Finally, we obtained 3-dimensional Shannon entropy.

### Mutual information

Mutual information can be regarded as the amount of information provided by one random variable about the other. In this work, mutual information is used to measure the information between consecutive bases and is defined as Formula (1):

1 $$I\left( {X,Y} \right) = \mathop \sum \limits_{{x\smallint {\mathcal{M}}}} \mathop \sum \limits_{{y \in {\mathcal{M}}}} P\left( {x,y} \right)\log_{2} \frac{{P\left( {x,y} \right)}}{P\left( x \right)P\left( y \right)}$$

where ${\mathcal{M}}$ is the set of nucleotides ${ }\left\{ {A,G,C,T} \right\}$ for each base pair ${ }\left( {x,y} \right)$,${\text{ P}}\left( {x,y} \right)$ is the joint probability, and ${\text{ P}}\left( x \right){\text{ and P}}\left( y \right)$ are the marginal probabilities. These probabilities are estimated based on the relative frequency in the corresponding gene sequence. $P\left( {x,y} \right)\log_{2} \frac{{P\left( {x,y} \right)}}{P\left( x \right)P\left( y \right)}$ is calculated and used as a feature. Therefore, a total of 17 MI-related features were calculated.

### Kullback‒Leibler divergence

Kullback‒Leibler divergence [48], also known as relative entropy, measures the difference between two probability distributions in the same event space and is defined as Formula (2):

2 $$D_{KL} = \mathop \sum \limits_{i} p\left( {x_{i} } \right)\log \frac{{p\left( {x_{i} } \right)}}{{q\left( {x_{i} } \right)}}$$

where $p\left({x}_{i}\right) and q\left({x}_{i}\right)$ are the probability distributions. The frequencies of nucleotides, dinucleotides, and trinucleotides in a given gene region sequence were compared with the corresponding frequencies in each gene sequence.

### Cross entropy

Cross entropy measures the difference information between a probability distribution $\mathrm{p}\left(x\right)$ and the other probability distribution $\mathrm{q}\left(x\right)$. It is calculated as Formula (3):

3 $$CE\left( {p,q} \right) = - \mathop \sum \limits_{i} p\left( {x_{i} } \right)\log \left( {q\left( {x_{i} } \right)} \right)$$

The frequencies of nucleotides, dinucleotides, and trinucleotides in a given gene region sequence were obtained from the corresponding frequencies in each gene sequence.

### Feature normalization

Due to the absence of some feature data of the source sequence, such as the Nc index values of some sequence misses, missing value processing methods (such as mean interpolation and homogeneous mean interpolation) were applied to complete the missing values. Moreover, each feature data usually has different dimensions and orders of magnitude. To ensure the reliability of the prediction results, the raw index data needed to be standardized.

All the feature vectors of instances were normalized according to the min–max formulation presented by Eq. (4):

4 $$x^{ * } = \frac{{x - x_{\min } }}{{x_{\max } - x_{\min } }}$$

where $x^{ * } \in [0,1]$, and $x_{\min }$ and $x_{\max }$, the minimum and maximum values of the features, respectively, denote the normalized value of $x^{ * }$.

### Evaluation metrics

In this study, we used accuracy, recall, precision, F-measure (F1), and G-mean to evaluate the predictive classification capabilities of the model. The ROC curve and AUC values were used to quantify the performance of the evaluation model [49].

The performance index formulas are as follows:

5 $$Accuracy = \frac{TP + TN}{{TP + FP + TN + FN}}$$

6 $$Precision = \frac{TP}{{TP + FP}}$$

7 $$Recall = \frac{TP}{{TP + FN}}$$

8 $$F - measure = \frac{2 \times Recall \times Precision}{{Recall + Precision}}$$

9 $$G - mean = \sqrt {Recall \times (1 - \frac{FP}{{FP + TN}})}$$

where TP, FP, TN, and FN are the numbers of true positives, false positives, true negatives, and false negatives, respectively.

### Cascaded BPNN model

A BPNN is a multilayer network consisting of an input layer, a hidden layer and an output layer. Among various classification algorithms, artificial neural networks (ANNs) have been proven to be effective algorithms that can be adapted to various research scenarios [50]. Among many ANN implementations, the backpropagation neural network (BPNN) is the most widely used because of its excellent function approximation capability. In the classification phase, BPNN only performs feedforward to achieve the final classification result. Although it is difficult to determine the optimal number of hidden layers and neurons for the classification task, a three-layer BPNN proves to be sufficient to fit the mathematical equations that approximate the mapping relationship between inputs and outputs. To make the classification results more accurate, we proposed a multilevel cascaded BPNN model.

**Fig. 2:**
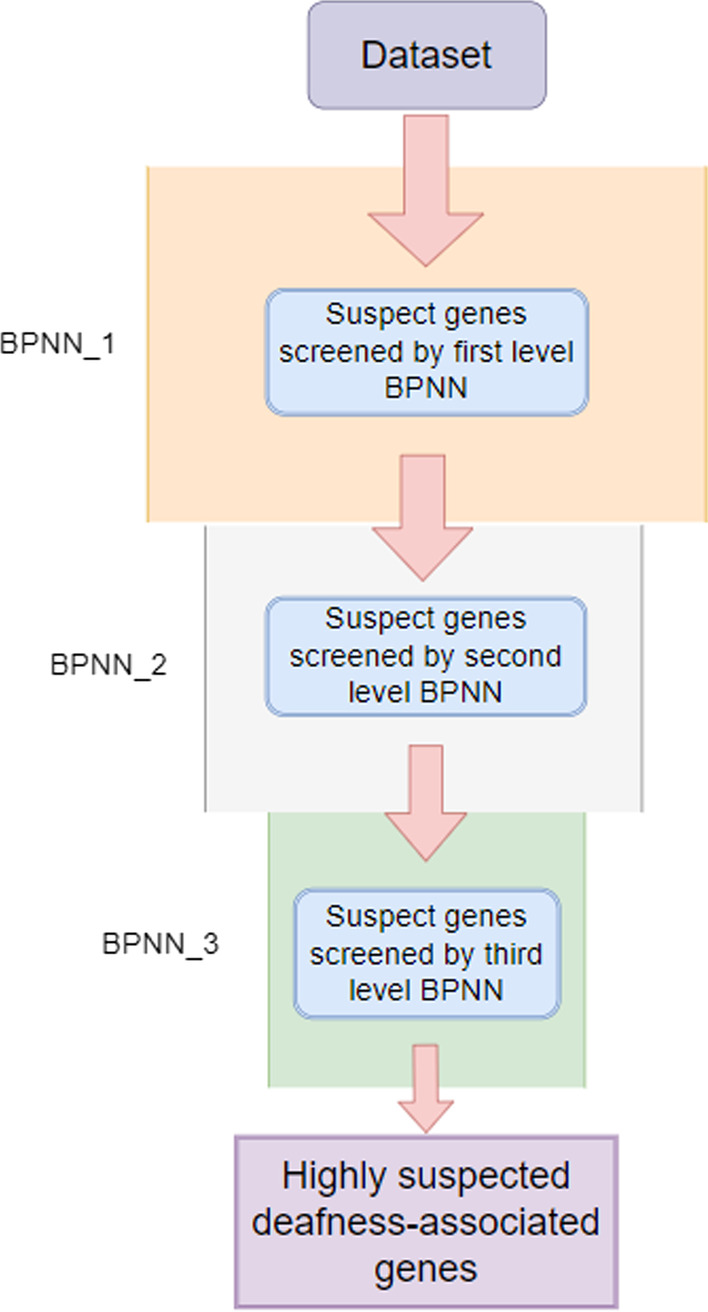
Cascaded BPNN model to predict deafness gene flow

We designed a three-level cascaded BPNN model to filter our data from coarse to fine. The model was first trained by inputting our extracted positive and negative sample features, while the parameters were tuned using the validation set. Then, our collated test dataset was fed into the first basic BPNN for prediction, during which our model was cycled 50 times, outputting the genes that were predicted positive each time. The more times a gene is predicted to be associated with a deafness gene, the more likely it is to be a candidate gene associated with deafness. If the gene is predicted in all 50 cycles of the experiment, then it is considered a highly suspicious deafness gene and is transferred to the next basic BPNN model for the experiment. A grid search method was used to find the optimal parameters, and finally, our candidate suspected deafness genes were obtained after a three-level BPNN screening. The contour diagram of the model is shown in Fig. 2:

A BPNN requires a set of predefined parameters to optimize the accuracy and generalization of the model. In this study, this experiment was implemented in Python 3.7. The BPNN package from the Scikit-Learn library in Python was called to build the model, and the parameters in the model were adjusted based on the experimental results.

Based on our validation experimental choices, we used a three-layer BPNN for cascading, and each level of the BPNN used a three-layer neural network model, i.e., one input layer, one hidden layer, and one output layer. According to the grid search method, we performed parameter search for the number of hidden layer neurons, activation function and learning rate. The number of hidden layer neurons ranged from 0 to 100 with a step size of 1, the learning rate was set as 0.001 to 0.1 with a step size of 0.001 and the activation functions were selected as Relu, Tanh and Logistic, respectively. The parameters of each level are shown in Table 3.

**Table 3:** Main parameter settings for the cascaded BPNN model of each layer

Layers	Number of hidden layer neurons	The activation function	Learning rate	Number of iterations	Momentum	Running time (s)
1	21	Relu	0.048	5000	0.8	1127.11
2	20	Tanh	0.048	5000	0.8	823.12
3	37	Logistic	0.098	5000	0.9	811.05

In the experiments, the dataset was divided into a training set, a validation set and a test set at a ratio of 6:2:2. According to Table 1, there were a total of 1140 samples (570 positive samples + 570 negative samples). The training and validation sets, including 912 samples, were randomly assigned in a ratio of 6:2 for model training and tuning. Then, the test set, consisting of 228 samples, was used to verify the validity of the model. After 50 replications, the mean values of six performance evaluation metrics, namely, accuracy, precision, recall, F1, G-mean and AUC, were calculated as shown in Table 4.

**Table 4:** Average of the performance evaluation metrics of the cascaded BP network classifier for 50 cycles

Model	Evaluation metrics					
	Accuracy	AUC	Precision	Recall	F1	G-mean
Cascaded BPNN	0.9463	0.9855	0.9781	0.9354	0.9559	0.9567

## Results

### Comparison of cascaded BPNN with other methods

**Fig. 3:**
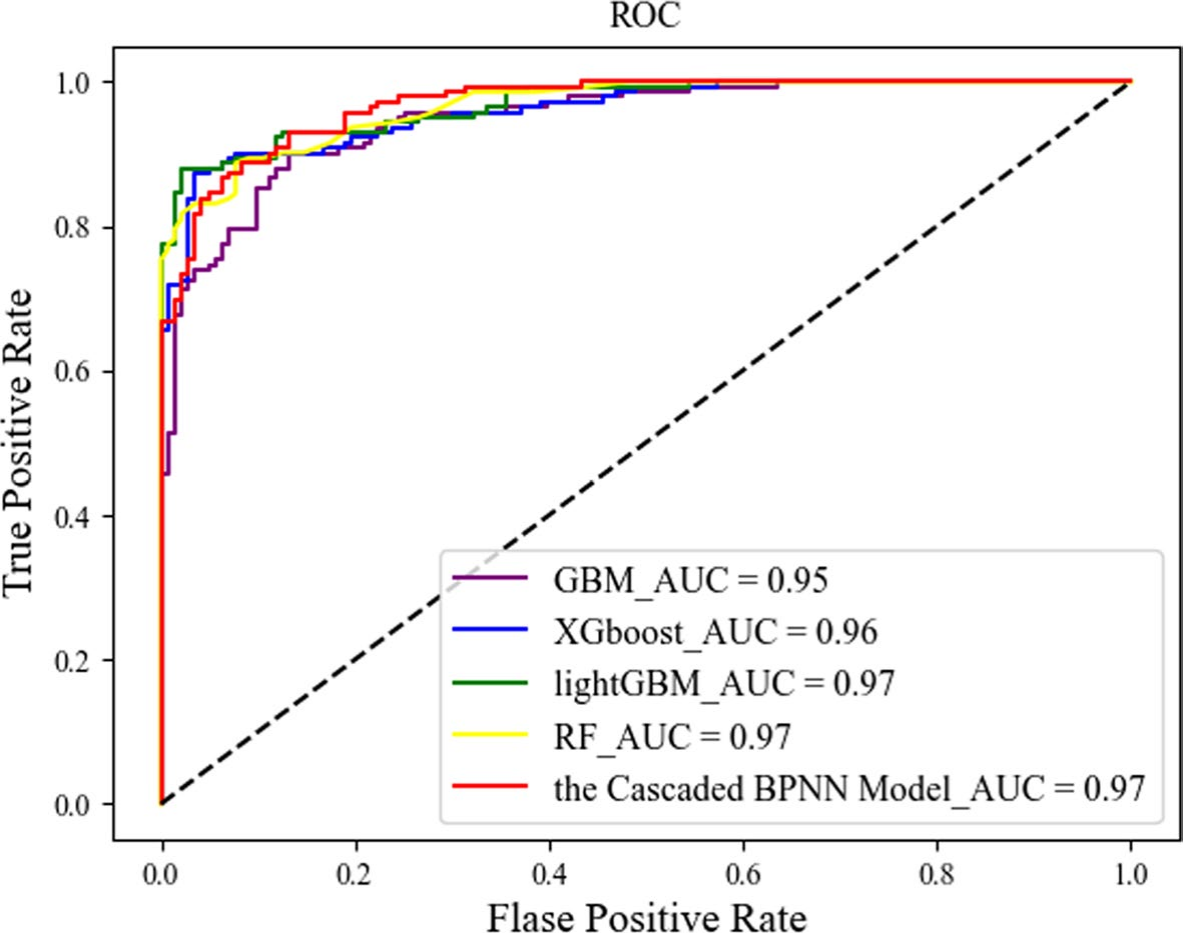
ROC curves for the different methods

**Fig. 4:**
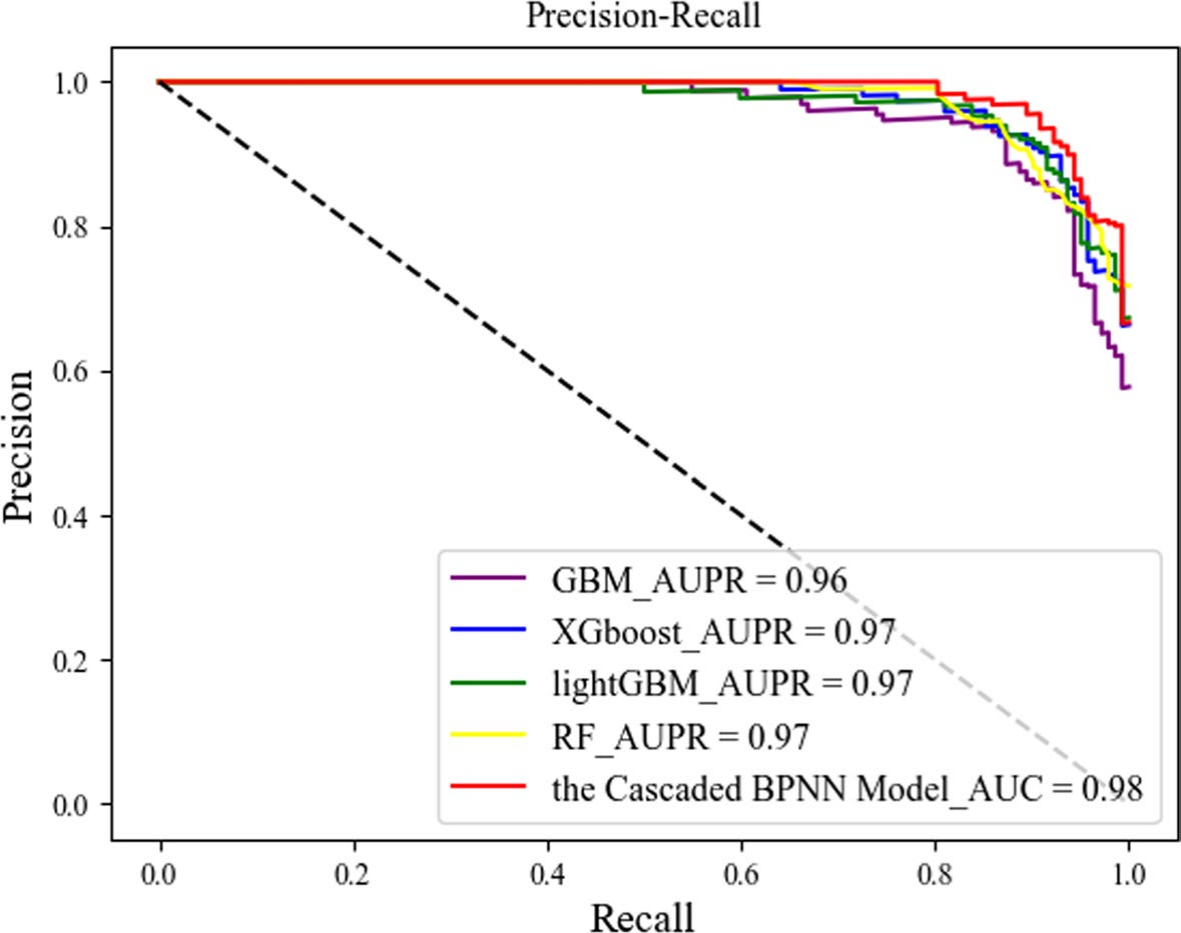
AUPR curves for the different methods

In this section, we compare our proposed cascaded algorithm with the more common currently used machine learning algorithms, including XGboost, GBM, lightGBM, and RF, trained and tested on the same training and test sets, and Figs. 3 and 4 show the ROC and AUPR curves obtained by each method.

We repeated the experiment 50 times. The larger the area under the curve, i.e., the higher the curve, the better the prediction performance of the corresponding algorithm. Figures 3 and 4 show the prediction performance of each classifier. From the figures, we can see that our proposed cascaded BPNN model had better AUC scores and a larger area under the curve than the other four machine learning models, and our cascaded model did not differ much from the other four models in terms of AUPR, showing the effectiveness of our cascaded model.

### Analysis of the prediction results

Finally, we used our cascaded model to analyze three test benchmark datasets (see Table 1). The tested genes were screened and ranked according to the corresponding number and score of genes predicted to be suspected deafness-related.

For test bench 1, we analyzed the data after 50 repeated runs using BPNN and the other four classifiers used previously. The results obtained are shown in Table 5.

**Table 5:** Prediction results for 45 genes in the literature

Model	Samples	Number of genes predicted to be positive
Cascaded BPNN	45	38
XGboost	45	36
lightGBM	45	36
GBM	45	34
RF	45	30

A gene may contain more than one gene CDS or protein sequence. A gene was identified as a candidate gene associated with deafness if both its CDS and corresponding protein sequence were predicted to be positive in 50 replicate experiments for each base classifier.

As shown in Table 4, the above predicted genes contained candidate deafness-associated sequences and so were identified as candidate deafness-associated genes by five classifiers, where our proposed cascaded BPNN model was able to identify the most deafness-associated genes among the five classifiers.

We identified five predicted highly suspected deafness-associated genes, namely, *COL1A1, GJC3, RRM2B, SALL4* and *SALL1*, in the available databases, including Ensemble [51] and OMIM [52], indicating that they were correctly identified as deafness-associated genes. Their corresponding annotations suggest that they are associated with deafness-related disorders. Site-specific mutations in *COL1A1, GJC3, RRM2B, SALL4,* and *SALL1* cause otosclerosis ([MIM:120150]), delayed hearing sensitivity ([MIM:611925]), sensorineural deafness ([MIM: 604712]), hearing loss ([MIM:607343]) and ear dysplasia ([MIM:602218]), among other deafness-related disorders. These disorders can lead to deafness or sensorineural deafness. These results suggest that our method is able to identify highly suspicious deafness-associated genes. The results for test bench 2 are shown in Table 6.

**Table 6:** Results of the predicted deafness genes for the three kit genes

Model	CDS number (times)	Protein sequence number (times*)*	Corresponding gene
cascaded BPNN	CCDS5746.1(50)	NP_000432(50*)*	*SLC26A4*
	CCDS9290.1(48)	NP_003995(48*)*	*GJB2*
	CCDS384.1(48)	NP_076872(48)	*GJB3*

*SLC26A4*, *GJB2*, and *GJB3* have gene CDSs and protein sequence fragments. They were identified as candidate deafness-associated genes by BPNN and four other classifiers, as shown in Table 5. Since these 3 genes are quite familiar and have been widely validated in the field of deafness research, the model correctly identified them as deafness-associated genes. These results demonstrated the ability of the proposed model to identify highly suspected deafness-associated genes.

Test bench 3 was used to further explore our model. The remaining 17,711 genes were screened and analyzed by the trained cascaded model. After three levels of screening, a total of 5013, 3577 and 1519 genes were screened from the first to the third levels, respectively. Among the 1519 genes finally screened, the suspected deafness-related genes were ranked according to the gene prediction scores. The 100 genes with the highest combined model prediction scores were considered highly suspected deafness-associated genes (see Additional file 1: Table A.1). We analyzed only the top 20 highly suspected deafness-associated genes (see Table 7).

**Table 7:** Top 20 genes predicted by our cascaded BPNN model

Gene names	References
*FSIP2*	
*SLC25A31*	
*GLRB*	Buerbank et al. [53]
*MEDAG*	
*ANO3*	Kunzelmann et al. [54]
*NUP153*	
*CENPS-CORT*	
*CD302*	
*YME1L1*	
*COG3*	
*NUP153*	
*TMEM242*	
*GAS7*	
*CNN1*	
*TTLL9*	
*SGTA*	
*RABL2A*	
*GRIA2*	Balaram et al. ^30^
*CPPED1*	
*YBX3*	

To gain insight into the accuracy of our estimator, despite the lack of an explicit classification of the unlabeled set, we downloaded a list of genes and diseases associated with hearing loss according to the text mining tools DISEASES [55] and DisGeNET [56]. We refer to these genes as deafness-associated genes (DAGs). The genes screened in the third screening were compared with deafness-associated genes, and more than 100 of the more than 1500 genes we screened could be found in the deafness-associated genes.

Three of the potential deafness-associated genes are reported in the available literature and databases. The third-ranked gene, GLRB, encodes a GlyRβ subunit associated with efferent olivocochlear innervation. The fifth-ranked gene, ANO3, is associated with impaired anoctamin function, which can lead to a wide range of disorders, such as hearing loss, bleeding disorders, ataxia and dystonia, persistent borrelia and mycobacterial infections, skeletal syndromes, such as jaw stem dysplasia and limb girdle muscular dystrophy, and cancer. The eighteenth-ranked gene, GRIA2, had significantly higher level content after 30 days of hearing loss. The other 17 highly suspected deafness-associated genes screened with our analytical model require further molecular biology studies.

The top 100 scored genes are listed in Additional file 1: Table A.1. A total of 20 of the genes have been reported in the literature and databases.

## Discussion

Computational prediction of deafness-associated genes is an important task for the diagnosis, treatment and prognosis of sudden deafness. A model for predicting highly suspected deafness-associated genes was constructed using a cascaded BPNN model based on a machine learning approach. In this paper, multiple sequence-based features are used. In the data processing step, we divide the dataset into a training set, a validation set and a test set. The training set is used to train the cascaded BPNN model, and the validation set is used to verify its parameters. In the predictions of the validation set, the average AUC of the cascaded BPNN model was 0.98 at each level, which was comparable to or better than the other four machine learning classifiers. In addition, three test sets were designed to further evaluate the accuracy and validity of the model, which included deafness-related genes collected in the literature, three genes from fifteen deafness-related gene mutation detection kits widely used in China, and the remaining 17,711 genes in the human genome.

In test bench 1, 40 of the 45 genes were highly suspected to be associated with deafness (Table 5). In test bench 2, all three genes recognized by the medical community to be associated with deafness were correctly predicted by the model (Table 6). Both test benches included genes with known labels, and the prediction results showed that the model proposed in this paper has good performance. In test bench 3, the data included the remaining 17,711 genes in the human genome, and three of the top 20 genes predicted by the model were found in related studies in the literature (Table 7). The related articles demonstrated that the identified genes are genes associated with deafness. Based on our results, the other 17 highly suspected deafness-associated genes need further molecular biology studies for identification. In addition, 23 other genes reported as deafness-associated genes in the literature and databases were among the top 100 scored genes of our results (Additional file 1: Table A.1).

The results of the analysis show the ability of our proposed model to help us to screen out highly suspected deafness-associated genes. By reducing the scope of data screening, this computational approach can save time and costs for biologists in deafness gene screening experiments. Moreover, it can provide necessary guidance for the clinical diagnosis and treatment of sudden deafness and help us further explore the associations of gene mutation loci with sudden deafness. In the follow-up research, the screened highly suspicious deafness-related genes by our experiment will be studied in clinical stage, to verify and search the association of these genes with sudden deafness by gene sequencing and other techniques.

## Supplementary Information

**Additional file 1**. Top 100 predictions of the cascaded BPNN model.

## Data Availability

The datasets and code are available at https://github.com/Cqerliu/Cascaded-BPNN-Model.

## References

[1] Cadoni G, Agostino S, Scipione S, Ippolito S, Caselli A, Marchese R, Paludetti G (2005). Sudden sensorineural hearing loss: our experience in diagnosis, treatment, and outcome. J Otolaryngol.

[2] Capaccio P, Ottaviani F, Cuccarini V, Bottero A, Schindler A, Cesana BM, Censuales S, Pignataro L (2007). Genetic and acquired prothrombotic risk factors and sudden hearing loss. Laryngoscope.

[3] Gross M, Wolf DG, Elidan J, Eliashar R (2007). Enterovirus, cytomegalovirus and Epstein-Barr virus infection screening in idiopathic sudden sensorineural hearing loss. Audiol Neurotol.

[4] de Oliveira Penido N, Ramos HVL, Barros FA, Cruz OLM, Toledo RN (2005). Clinical, etiological and progression factors of hearing in sudden deafness. Braz J Otorhinolar.

[5] Salahaldin AH, Bener A, Elhakeem AAM, Abdulhadi K (2004). Management of idiopathic sudden sensorineural hearing loss: experience in newly developing Qatar. Int Tinnitus J.

[6] Byl FM (1997). Seventy-six cases of presumed sudden hearing loss occurring in 1973: prognosis and incidence. Laryngoscope.

[7] Nosrati-Zarenoe R, Arlinger S, Hultcrantz E (2007). Idiopathic sudden sensorineural hearing loss: results drawn from the Swedish national database. Acta Oto-Laryngol.

[8] Byl FM (1984). Sudden hearing loss: eight years’ experience and suggested prognostic table. Laryngoscope.

[9] Chen K, Sun L, Zong L, Wu X, Zhan Y, Dong C, Cao H, Tang H, Jiang H (2016). GJB2 and mitochondrial 12S rRNA susceptibility mutations in sudden deafness. Eur Arch Oto-Rhino-L.

[10] Gross M, Friedman G, Eliashar R, Koren-Morag N, Goldschmidt N, Atta IA, Ben-Yehuda A (2006). Impact of methionine synthase gene and methylenetetrahydrofolate reductase gene polymorphisms on the risk of sudden sensorineural hearing loss. Audiol Neurotol.

[11] Uchida Y, Sugiura S, Ando F, Shimokata H, Nakashima T (2010). Association of the C677T polymorphism in the methylenetetrahydrofolate reductase gene with sudden sensorineural hearing loss. Laryngoscope.

[12] Hamidi AK, Yazdani N, Seyedjavadi KH, Ahrabi NZ, Tajdini A, Aghazadeh K, Amoli MM (2019). MTHFR AND ApoE genetic variants association with sudden sensorineural hearing loss. Am J Otolaryngol.

[13] Furuta T, Teranishi M, Uchida Y, Nishio N, Kato K, Otake H, Yoshida T, Tagaya M, Suzuki H, Sugiura M, Sone M, Hiramatsu M, Sugiura S, Ando F, Shimokata H, Nakashima T (2011). Association of interleukin-1 gene polymorphisms with sudden sensorineural hearing loss and Ménière’s disease. Int J Immunogenet.

[14] Yang C, Hwang C, Yang M, Lin P, Chuang J (2015). Expression of toll-like receptor genes in leukocytes of patients with sudden sensorineural hearing loss. Laryngoscope.

[15] Cao Z, Gao J, Huang S, Xiang H, Zhang C, Zheng B, Zhan X, Chen R, Chen B (2019). Genetic polymorphisms and susceptibility to sudden sensorineural hearing loss: a systematic review. Audiol Neurotol.

[16] Márton K, Uzsaly J, Bodzai G, Harmat K, Németh A, Gerlinger I, Bakó P (2019). Analysis of prognostic factors influencing the effectiveness of treatment in sudden sensorineural hearing loss. Orv Hetil.

[17] Delgado-Gil JE, Krstulovic C, Pérez-Guillén V, García-Zamora E, Pérez-Garrigues H (2019). Sordera súbita idiopática. Revisión de 58 casos. Revista ORL.

[18] Ječmenica J, Bajec-Opančina A (2014). Sudden hearing loss in children. Clin Pediatr.

[19] Rossini B, Penido N, Munhoz M, Bogaz E, Curi R (2017). Sudden sensorioneural hearing loss and autoimmune systemic diseases. Int Arch Otorhinolaryngol.

[20] Kuhn M, Heman-Ackah SE, Shaikh JA, Roehm PC (2011). Sudden sensorineural hearing loss. Trends Amplif.

[21] Yu H, Li H (2018). Association of vertigo with hearing outcomes in patients with sudden sensorineural hearing loss a systematic review and meta-analysis. JAMA Otolaryngol Head Neck Surg.

[22] Uchida Y, Teranishi M, Nishio N, Sugiura S, Hiramatsu M, Suzuki H, Kato K, Otake H, Yoshida T, Tagaya M, Suzuki H, Sone M, Ando F, Shimokata H, Nakashima T (2013). Endothelin-1 gene polymorphism in sudden sensorineural hearing loss. Laryngoscope.

[23] Imtiaz A, Kohrman DC, Naz S (2014). A frameshift mutation in GRXCR2 causes recessively inherited hearing loss. Hum Mutat.

[24] Cadoni G, Gaetani E, Picciotti PM, Arzani D, Quarta M, Giannantonio S, Paludetti G, Boccia S (2015). A case–control study on proinflammatory genetic polymorphisms on sudden sensorineural hearing loss. Laryngoscope.

[25] Carter H, Douville C, Stenson PD, Cooper DN, Karchin R (2013). Identifying Mendelian disease genes with the variant effect scoring tool. BMC Genom.

[26] Lê Cao K, Boitard S, Besse P (2011). Sparse PLS discriminant analysis biologically relevant feature selection and graphical displays for multiclass problems. BMC Bioinform.

[27] Xiao Q, Luo J, Liang C, Cai J, Ding P (2018). A graph regularized non-negative matrix factorization method for identifying microRNA-disease associations. Bioinformatics.

[28] Azadifar S, Rostami M, Berahmand K, Moradi P, Oussalah M (2022). Graph-based relevancy-redundancy gene selection method for cancer diagnosis. Comput Biol Med.

[29] Saberi-Movahed F, Rostami M, Berahmand K, Karami S, Tiwari P, Oussalah M, Band SS (2022). Dual Regularized unsupervised feature selection based on matrix factorization and minimum redundancy with application in gene selection. Knowl Based Syst.

[30] Balaram P, Hackett TA, Polley DB (2019). Synergistic transcriptional changes in AMPA and GABAA receptor genes support compensatory plasticity following unilateral hearing loss. Neuroscience.

[31] Guo Y, Liu S, Li Z, Shang X (2018). BCDForest: a boosting cascade deep forest model towards the classification of cancer subtypes based on gene expression data. BMC Bioinform.

[32] Bing D, Ying J, Miao J (2018). Predicting the hearing outcome in sudden sensorineural hearing loss via machine learning models. Clin Otolaryngol.

[33] Nayak DR, Zhang Y, Das DS, Panda S (2019). MJaya-ELM: a Jaya algorithm with mutation and extreme learning machine based approach for sensorineural hearing loss detection. Appl Soft Comput.

[34] Azaiez H, Booth KT, Ephraim SS, Crone B, Black-Ziegelbein EA, Marini RJ, Shearer AE, Sloan-Heggen CM, Kolbe D, Casavant T, Schnieders MJ, Nishimura C, Braun T, Smith RJH (2018). Genomic landscape and mutational signatures of deafness-associated genes. Am J Hum Genet.

[35] Davoli T, Xu AW, Mengwasser KE, Sack LM, Yoon JC, Park PJ, Elledge SJ (2013). Cumulative haploinsufficiency and triplosensitivity drive aneuploidy patterns and shape the cancer genome. Cell.

[36] Perez-Iratxeta C, Bork P, Andrade MA (2002). Association of genes to genetically inherited diseases using data mining. Nat Genet.

[37] Freudenberg J, Propping P (2002). A similarity-based method for genome-wide prediction of disease-relevant human genes. Bioinformatics.

[38] El MAV, Cuelenaere K, Kemmeren PP, Leunissen JAM, Brunner HG (2003). A new web-based data mining tool for the identification of candidate genes for human genetic disorders. Eur J Hum Genet.

[39] Turner FS, Clutterbuck DR (2003). Semple, C.A.M. POCUS: mining genomic sequence annotation to predict disease genes. Genome Biol.

[40] Tiffin N, Kelso JF, Powell AR, Pan H, Bajic VB, Hide WA (2005). Integration of text- and data-mining using ontologies successfully selects disease gene candidates. Nucleic Acids Res.

[41] Aerts S, Lambrechts D, Maity S, Van Loo P, Coessens B, De Smet F, Tranchevent L, De Moor B, Marynen P, Hassan B, Carmeliet P, Moreau Y (2006). Gene prioritization through genomic data fusion. Nat Biotechnol.

[42] Franke L, Van Bakel H, Fokkens L, De Jong ED, Egmont-Petersen M, Wijmenga C (2006). Reconstruction of a functional human gene network, with an application for prioritizing positional candidate genes. Am J Hum Genet.

[43] Sharp PM, Bailes E, Grocock RJ, Peden JF, Sockett RE (2005). Variation in the strength of selected codon usage bias among bacteria. Nucleic Acids Res.

[44] Rice P, Longden I, Bleasby A (2000). EMBOSS: the European molecular biology open software suite. Trends Genet.

[45] Krogh A, Larsson B, von Heijne G, Sonnhammer ELL (2001). Predicting transmembrane protein topology with a hidden markov model: application to complete genomes11Edited by F. Cohen J Mol Biol.

[46] Team, R.D.C. A language and environment for statistical computing. Vienna, Austria: R Foundation for Statistical Computing. 2014.

[47] Shannon CE (1948). A mathematical theory of communication. Bell Syst Tech J.

[48] Kullback S, Leibler R (1951). On information and sufficiency. Ann Math Stat.

[49] Fawcett T (2006). An introduction to ROC analysis. Pattern Recogn Lett.

[50] Liu Y, Jing W, Xu L (2016). Parallelizing backpropagation neural network using mapreduce and cascading model. Comput Intell Neurosci.

[51] Ensembl is based at the European Molecular Biology Laboratory’s European Bioinformatics Institute(EMBL-EBI), located on the Wellcome Genome Campus in Hinxton, south of the city of Cambridge, United Kingdom. http://www.ensembl.org/index.html?redirect=no. Accessed 18 July 2020.

[52] OMIM (Online Mendelian inheritance in man). Baltimore: Johns Hopkins University, Center for Medical Genetics. 1996. http://www.ncbi.nlm.nih.gov/omim/. Accessed 3 Feb 2021.

[53] Buerbank S, Becker K, Becker C, Brandt N, Engel J, Knipper M, Schick B, Dlugaiczyk J (2011). Developmental regulation of glycine receptors at efferent synapses of the murine cochlea. Histochem Cell Biol.

[54] Balint B, Bhatia KP (2014). Dystonia: an update on phenomenology, classification, pathogenesis and treatment. Curr Opin Neurol.

[55] Pletscher-Frankild S, Palleja A, Tsafou K, Binder JX, Jensen LJ (2015). DISEASES: text mining and data integration of disease-gene associations. Methods.

[56] Pinero J, Queralt-Rosinach N, Bravo A, Deu-Pons J, Bauer-Mehren A, Baron M, Sanz F, Furlong LI..DisGeNET: a discovery platform for the dynamical exploration of human diseases and their genes. Database (Oxford).2015:bav028. https://diseases.jensenlab.org/. 10.1093/database/bav028 PMC4397996 25877637

