# Supplementary material for: Deafness gene screening based on a multilevel cascaded BPNN model
Source: BMC Bioinformatics. 2023 Feb 20;24:56. doi: 10.1186/s12859-023-05182-7 (PMC9942297; doi:10.1186/s12859-023-05182-7)
Supplement: Supplementary file 1 — Additional file 1. Top 100 predictions of the cascaded BPNN model. [file 12859_2023_5182_MOESM1_ESM.docx]

# Appendix A. Additional results

In this appendix, we show the top 100 scored genes of test bench dataset 3 in **Table** **A.1**.

**Table A.1** Top 100 predictions of the cascaded BPNN model.

| **Ranking of predicted results** | **Gene name** |  | **Reference** |
| --- | --- | --- | --- |
| 1 | *FSIP2* |  |  |
| 2 | *SLC25A31* |  |  |
| 3 | *GLRB* | ******* | [1]. Buerbank, S. and K. Becker, et al. (2011). "Developmental regulation of glycine receptors at efferent synapses of the murine cochlea." Histochemistry and Cell Biology 136(4): 387-398. |
| 4 | *MEDAG* |  |  |
| 5 | *ANO3* | ******* | [2]. Balint, B. and K. P. Bhatia(2014). "Dystonia: An update on phenomenology, classification, pathogenesis and treatment." Current opinion in neurology 27(4): 468-476. |
| 6 | *NUP153* |  |  |
| 7 | *CENPS-CORT* |  |  |
| 8 | *CD302* |  |  |
| 9 | *YME1L1* |  |  |
| 10 | *COG3* |  |  |
| 11 | *TMEM242* |  |  |
| 12 | *GAS7* |  |  |
| 13 | *CNN1* |  |  |
| 14 | *TTLL9* |  |  |
| 15 | *SGTA* |  |  |
| 16 | *RABL2A* |  |  |
| 17 | *GRIA2* | ******* | [3]. Balaram, P. and T. A. Hackett, et al. (2019). "Synergistic Transcriptional Changes in AMPA and GABAA Receptor Genes Support Compensatory Plasticity Following Unilateral Hearing Loss." Neuroscience 407: 108-119. |
| 18 | *RIPPLY2* |  |  |
| 19 | *CPPED1* |  |  |
| 20 | *YBX3* |  |  |
| 21 | *CRYGN* | ******* | [4]. Hartwich, H. and E. Rosengauer, et al. (2016). "Functional Role of γ-Crystallin N in the Auditory Hindbrain." PLOS ONE 11(8): e0161140. |
| 22 | *PID1* |  |  |
| 23 | *VPS13A* |  |  |
| 24 | *EYS* |  |  |
| 25 | *ABCA13* | ******* | [5]. Mothe, A. J. and I. R. Brown(2001). "Expression of mRNA encoding extracellular matrix glycoproteins SPARC and SC1 is temporally and spatially regulated in the developing cochlea of the rat inner ear." Hearing research 155(1): 161-174. |
| 26 | *PURG* |  |  |
| 27 | *AIPL1* |  |  |
| 28 | *SPARC* | ******* | [6]. Hamdan, N. and C. Mehawej, et al. (2020). "A homozygous stop gain mutation in BOD1 gene in a Lebanese patient with syndromic intellectual disability." Clinical Genetics 98(3): 288-2 |
| 29 | *TRIAP1* |  |  |
| 30 | *SPATA33* |  |  |
| 31 | *BOD1* | ******* | [7]. Mansour, S. L. and C. Li, et al. (2013). "Genetic rescue of Muenke syndrome model hearing loss reveals prolonged FGF-dependent plasticity in cochlear supporting cell fates." Genes & Development 27(21): 2320-2331. |
| 32 | *PCDH10* |  |  |
| 33 | *NTMT1* |  |  |
| 34 | *FGF8* | ******* | [8]. LUO, Y. U. and F. KONG, et al. (2014). "Loss of ASAP3 destabilizes cytoskeletal protein ACTG1 to suppress cancer cell migration." Molecular medicine reports 9(2): 387-394. |
| 35 | *ATP5ME* |  |  |
| 36 | *HIST1H2BC* |  |  |
| 37 | *MSH3* |  |  |
| 38 | *ASAP3* | ******* | [9]. Perl, E. and P. Ravisankar, et al. (2022). "Stx4 is required to regulate cardiomyocyte Ca2+ handling during vertebrate cardiac development." Human Genetics and Genomics Advances 3(3): 100115. |
| 39 | *CLTA* |  |  |
| 40 | *VPS13C* |  |  |
| 41 | *STX4* | ******* | [10]. Hoshino, T. and K. Tabuchi, et al. (2008). "The Non-steroidal Anti-inflammatory Drugs Protect Mouse Cochlea against Acoustic Injury." Tohoku journal of experimental medicine 216(1): 53-59. |
| 42 | *UCK1* |  |  |
| 43 | *LOX* | ******* | [11]. Pang, J. and H. Xiong, et al. (2019). "SIRT1 protects cochlear hair cell and delays age-related hearing loss via autophagy." Neurobiology of Aging 80: 127-137. |
| 44 | *SHOX2* |  |  |
| 45 | *SIRT1* | ******* | [12]. Scott, D. and V. Jordan, et al. (2015). "1p36 deletion syndrome: an update." The Application of Clinical Genetics 8: 189-200. |
| 46 | *ZNF292* |  |  |
| 47 | *KHDC3L* |  |  |
| 48 | *ECE1* | ******* | [13]. Darvish, H. and S. Esmaeeli-Nieh, et al. (2010). "A clinical and molecular genetic study of 112 Iranian families with primary microcephaly." Journal of Medical Genetics 47(12): 823-828. |
| 49 | *ASPM* | ******* | [14]. Yousaf, R. and Q. Meng, et al. (2015). "MAP3K1 function is essential for cyto-architecture of mouse organ of Corti and survival of auditory hair cells." Disease Models & Mechanisms 8: 1543-1553. |
| 50 | *FGF8* | ******* | [15]. Keppler-Noreuil, K. M. and A. J. Carroll, et al. (1998). "Chromosome 18q paracentric inversion in a family with mental retardation and hearing loss." American journal of medical genetics 76(5): 372-378. |
| 51 | *CALM3* |  |  |
| 52 | *HAUS8* |  |  |
| 53 | *MBP* | ******* | [16]. Keppler-Noreuil, K. M. and A. J. Carroll, et al. (1998). "Chromosome 18q paracentric inversion in a family with mental retardation and hearing loss." American journal of medical genetics 76(5): 372-378. |
| 54 | *GOLM1* |  |  |
| 55 | *FAM107A* |  |  |
| 56 | *EDF1* |  |  |
| 57 | *PDGFB* |  |  |
| 58 | *SMARCD3* |  |  |
| 59 | *RBM8A* |  |  |
| 60 | *HOXC10* |  |  |
| 61 | *CNN2* |  |  |
| 62 | *ATCAY* |  |  |
| 63 | *SLC4A4* | ******* | [17]. Grandi, F. C. and L. De Tomasi, et al. (2020). "Single-Cell RNA Analysis of Type I Spiral Ganglion Neurons Reveals a Lmx1a Population in the Cochlea." Frontiers in Molecular Neuroscience 13. |
| 64 | *DHODH* |  |  |
| 65 | *TMEM87A* |  |  |
| 66 | *RANBP2* |  |  |
| 67 | *FCMR* |  |  |
| 68 | *PHACTR3* |  |  |
| 69 | *TMCC2* |  |  |
| 70 | *PNMA8A* |  |  |
| 71 | *PRM2* |  |  |
| 72 | *C11orf98* |  |  |
| 73 | *DYNC2H1* |  |  |
| 74 | *USF2* | ******* | [18]. Singer, W. and R. Panford-Walsh, et al. (2008). "Salicylate alters the expression of calcium response transcription factor 1 in the cochlea: Implications for brain-derived neurotrophic factor transcriptional regulation." Molecular pharmacology 73(4): 1085-1091. |
| 75 | *GAS8* |  |  |
| 76 | *GTF3C5* |  |  |
| 77 | *MAP2K7* |  |  |
| 78 | *OAZ1* |  |  |
| 79 | *RBMXL3* |  |  |
| 80 | *CCDC86* |  |  |
| 81 | *PTGES3L-AARSD1* |  |  |
| 82 | *DRAP1* |  |  |
| 83 | *BEX4* |  |  |
| 84 | *NCALD* |  |  |
| 85 | *RPL35* |  |  |
| 86 | *KRT6A* |  |  |
| 87 | *SH3GL1* |  |  |
| 88 | *EVC* | ******* | [19]. Sivakumaran, T. A. and M. M. Lesperance(2004). "Haplotype and linkage disequilibrium analysis of the CRMP1 and EVC genes." INTERNATIONAL JOURNAL OF MOLECULAR MEDICINE 14(5): 903-907. |
| 89 | *BORCS8* |  |  |
| 90 | *SZRD1* |  |  |
| 91 | *DNAJB1* |  |  |
| 92 | *RPL19* |  |  |
| 93 | *GIMAP6* |  |  |
| 94 | *VEGFA* | ******* | [20]. Asgarbeik, S. and A. Vahidi, et al. (2021). "VEGFA gene haplotypes in Meniere's disease." Gene Reports 24: 101244. |
| 95 | *GRIPAP1* |  |  |
| 96 | *C11orf97* |  |  |
| 97 | *DMKN* |  |  |
| 98 | *LRP1B* |  |  |
| 99 | *ANXA8* |  |  |
| 100 | *HLA-F* |  |  |

Genes marked with an asterisk (*) are those related to deafness mentioned in the Web of Science and the corresponding references.
